# Author Correction: Integration of human adipocyte chromosomal interactions with adipose gene expression prioritizes obesity-related genes from GWAS

**DOI:** 10.1038/s41467-018-05849-3

**Published:** 2018-08-22

**Authors:** David Z. Pan, Kristina M. Garske, Marcus Alvarez, Yash V. Bhagat, James Boocock, Elina Nikkola, Zong Miao, Chelsea K. Raulerson, Rita M. Cantor, Mete Civelek, Craig A. Glastonbury, Kerrin S. Small, Michael Boehnke, Aldons J. Lusis, Janet S. Sinsheimer, Karen L. Mohlke, Markku Laakso, Päivi Pajukanta, Arthur Ko

**Affiliations:** 1 Department of Human Genetics, David Geffen School of Medicine at UCLA, Los Angeles, CA 90095 USA; 2 Bioinformatics Interdepartmental Program, UCLA, Los Angeles, CA 90095 USA; 3 Department of Genetics, University of North Carolina, Chapel Hill, NC 27599 USA; 4 Department of Biomedical Engineering, University of Virginia, Charlottesville, VA 22904 USA; 5 Big Data Institute, University of Oxford, Oxford, OX3 7LF UK; 6 Department of Twin Research and Genetic Epidemiology, King’s College, London, UK; 7 Department of Biostatistics, University of Michigan, Ann Arbor, MI 48109 USA; 8 Department of Biomathematics, David Geffen School of Medicine at UCLA, Los Angeles, CA 90095 USA; 9 Institute of Clinical Medicine, Internal Medicine, University of Eastern Finland and Kuopio University Hospital, Kuopio, FI-70210 Finland; 10 Molecular Biology Institute at UCLA, Los Angeles, CA 90095 USA

Correction to: *Nature Communications* 10.1038/s41467-018-03554-9, published online 17 April 2018

In the original version of this Article, Supplementary Table 10 contained incorrect primer sequences for the mobility shift assay for SNP rs4776984. These errors have now been fixed and the corrected version of the Supplementary Information PDF is available to download from the HTML version of the Article.

