# Supplementary material for: Integration of human adipocyte chromosomal interactions with adipose gene expression prioritizes obesity-related genes from GWAS
Source: Nat Commun. 2018 Apr 17;9:1512. doi: 10.1038/s41467-018-03554-9 (PMC5904163; doi:10.1038/s41467-018-03554-9)
Supplement: Supplementary file 1 — Supplementary Information [file 41467_2018_3554_MOESM1_ESM.pdf]

Integration of human adipocyte chromosomal interactions with adipose gene expression prioritizes obesity-related genes from GWAS

Pan et al.

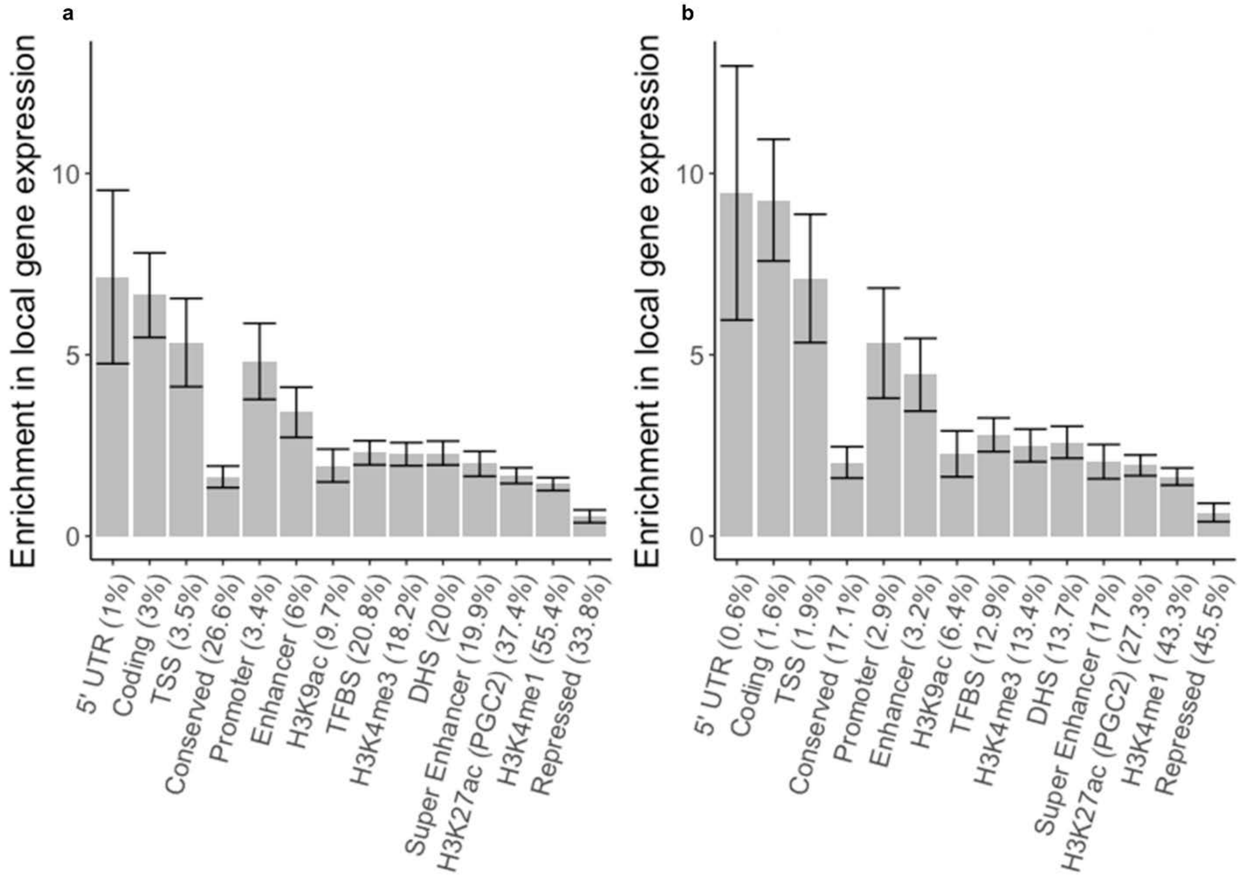

Supplementary Figure 1. Modification to LD Score regression software does not show significant changes when compared with the data obtained using the published version.

Enrichments in local gene expression with error bars for different categories using the LD score regression analysis. For the horizontal axis labels, the value in parentheses shows the percentage of SNPs contained within the respective annotation category that contributed to the enrichment calculation (for the full data on all 52 baseline annotation categories, see Supplementary Table 3-4). Error bars represent jackknife standard errors around the estimates of enrichment. (a) Enrichment in local gene expression for the modified LD Score regression software. (b) Enrichment in local gene expression for the original, unmodified LD Score regression software.

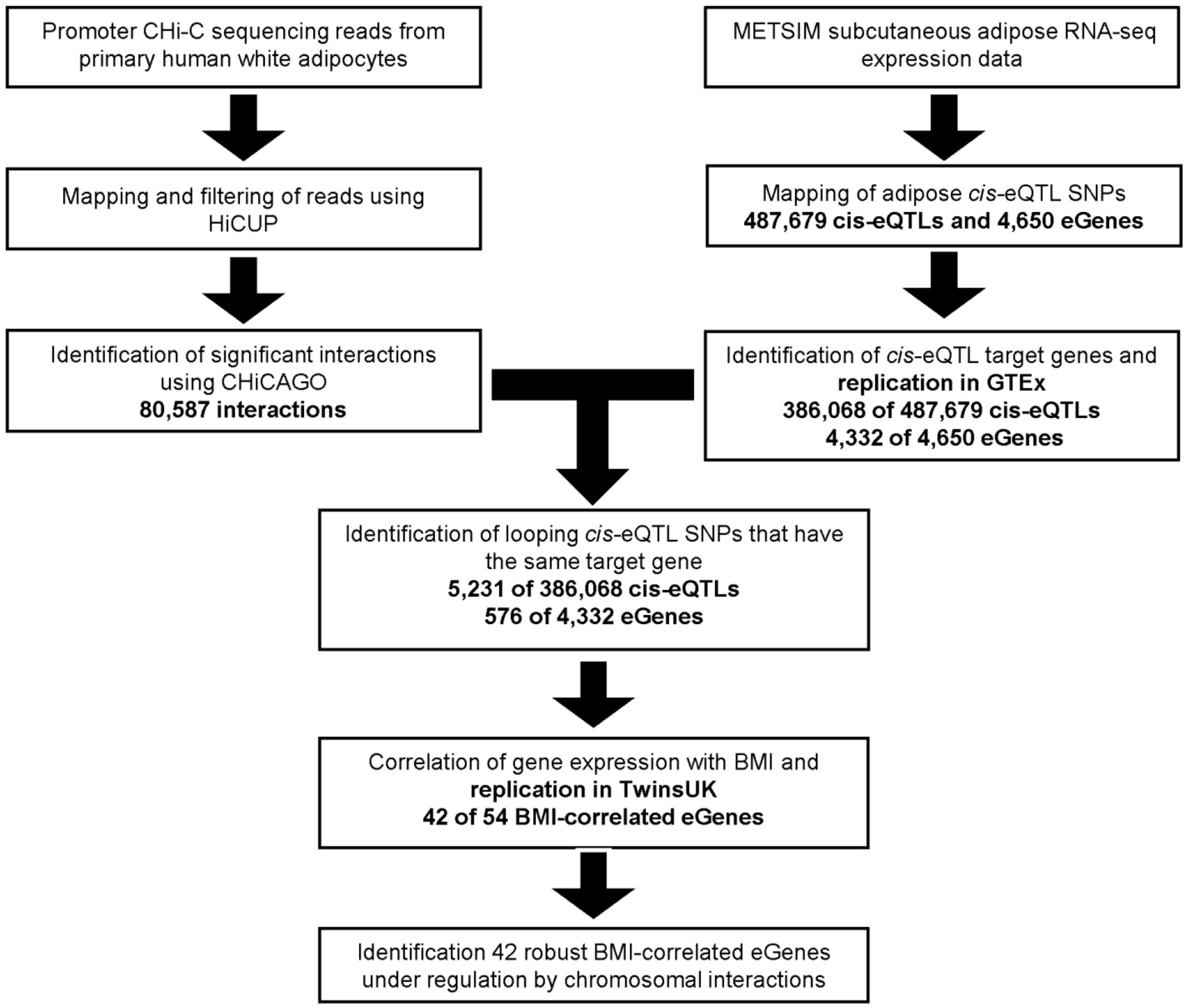

Supplementary Figure 2. Overview of the study design targeted to identify causal and reactive BMI-correlated genes.

Flow chart showing the data processing and analysis pipeline of the promoter Capture Hi-C in primary human white adipocytes (HWA) (the left side); adipose RNA-sequencing followed by *cis*-eQTL mapping (the right side); and the integration of these genomics data (in the middle) to identify eGenes correlated with BMI.

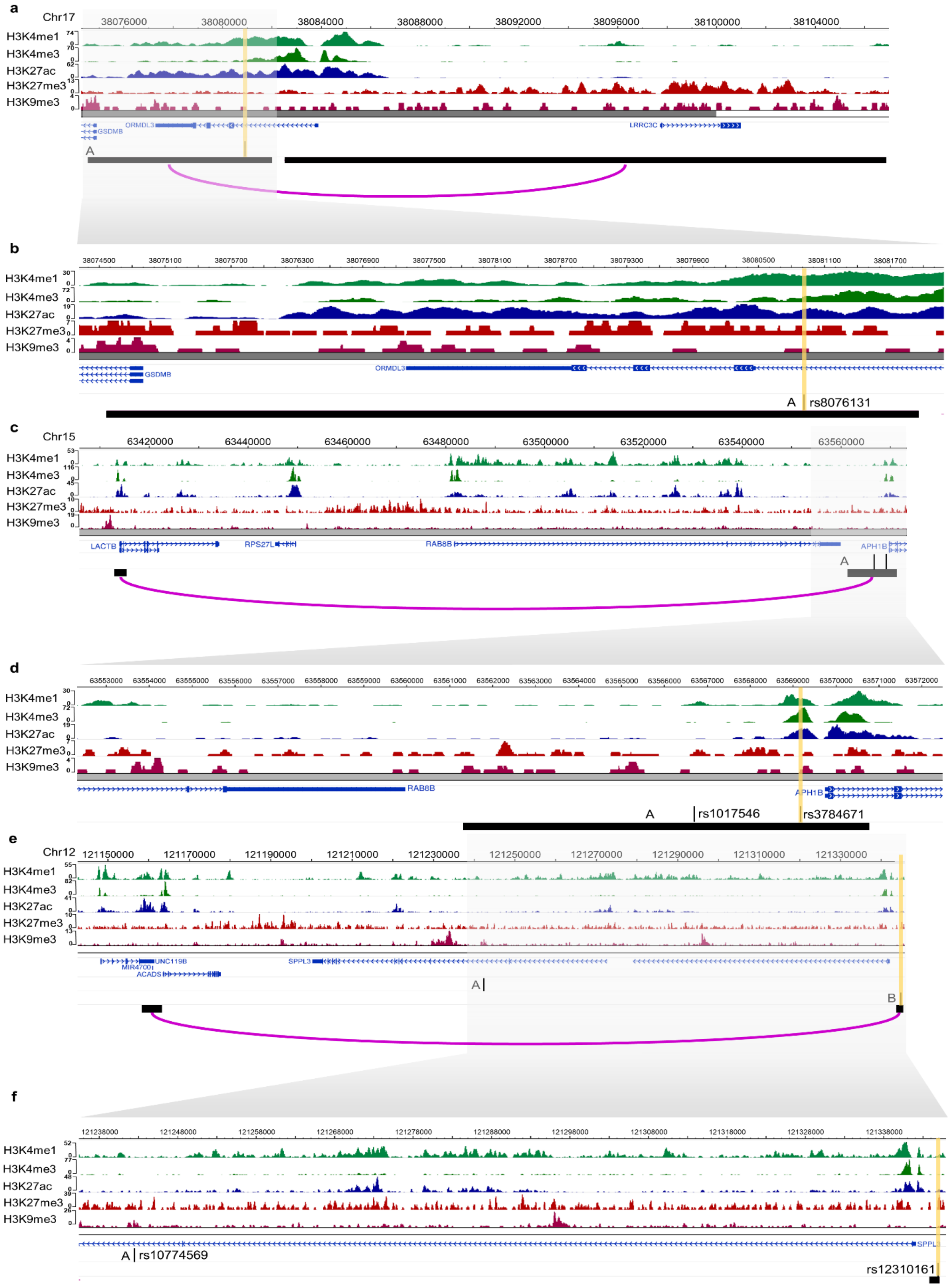

a
Chr17
38076000
38080000
38084000
38088000
38092000
38096000
38100000
38104000
H3K4me1
H3K4me3
H3K27ac
H3K27me3
H3K9me3
ORMDL3
GSDMB
LRRC3C
A
b
38074500
38075100
38075700
38076300
38076900
38077500
38078100
38078700
38079300
38079900
38080500
38081100
38081700
H3K4me1
H3K4me3
H3K27ac
H3K27me3
H3K9me3
GSDMB
ORMDL3
A
rs8076131
c
Chr15
63420000
63440000
63460000
63480000
63500000
63520000
63540000
63560000
H3K4me1
H3K4me3
H3K27ac
H3K27me3
H3K9me3
LACTB
RPS27L
RAB8B
APH1B
A
d
63553000
63554000
63555000
63556000
63557000
63558000
63559000
63560000
63561000
63562000
63563000
63564000
63565000
63566000
63567000
63568000
63569000
63570000
63571000
63572000
H3K4me1
H3K4me3
H3K27ac
H3K27me3
H3K9me3
RAB8B
APH1B
A
rs1017546
rs3784671
e
Chr12
121150000
121170000
121190000
121210000
121230000
121250000
121270000
121290000
121310000
121330000
H3K4me1
H3K4me3
H3K27ac
H3K27me3
H3K9me3
UNC119B
MIR4700
ACADS
SPPL3
A
B
f
121238000
121248000
121258000
121268000
121278000
121288000
121298000
121308000
121318000
121328000
121338000
H3K4me1
H3K4me3
H3K27ac
H3K27me3
H3K9me3
SPPL3
A
rs10774569
rs12310161

Supplementary Figure 3. Promoter Capture Hi-C enables refinement of the GWAS loci that colocalizes with *cis*-eQTLs interacting with the target gene promoter of *ORMDL3*, *LACTB*, and *ACADS*.

Genomic landscape of the lipid GWAS locus, *ORMDL3* (panels a, b), metabolite GWAS locus, *LACTB* (panels c, d), and metabolite GWAS locus, *ACADS* (panels e, f), modified from the WashU Genome Browser to show the histone mark calls from ChIP-seq data; gene transcripts; promoter and eQTL *Hin*dIII fragments that interact in primary human white adipocytes (HWA); and GWAS SNP (A, the rs number indicated in the magnified box) or their LD proxies if applicable (B, r^2>0.80) located in the interacting *Hin*dIII fragment. The vertical yellow band highlights the significantly influential variant (the rs number is indicated in the magnified box). (a) Genomic landscape containing *ORMDL3* and the interacting lipid GWAS SNP. (b) Magnification of the boxed region in (a). (c) Genomic landscape containing *LACTB* and the interacting metabolite GWAS SNPs. (d) Magnification of the boxed region in (c). (e) Genomic landscape containing *ACADS* and the interacting *cis*-eQTLs and corresponding metabolite GWAS SNP. (f) Magnification of the boxed region in (e).

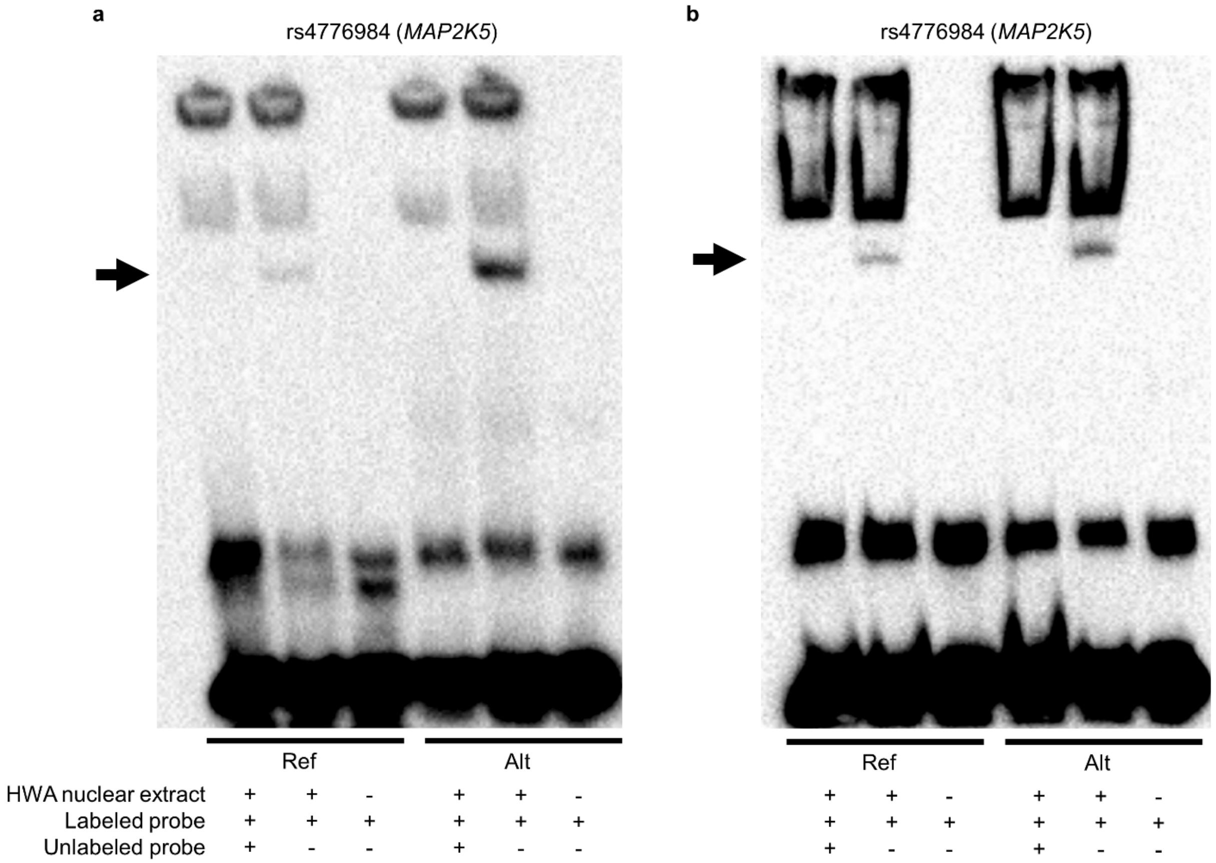

Supplementary Figure 4. Two independent replicates for the Electrophoretic mobility shift assay (EMSA) data show increased binding of nuclear protein extracted from primary human white adipocytes (HWA) to the alternate allele when compared to the reference allele of the *MAP2K5 cis*-eQTL SNP rs4776984.

Biotinylated (labeled probe) 31-bp oligonucleotide complexes with +/-15 bp flanking the reference or alternate allele for variant rs4776984 were incubated with nuclear protein extracted from primary HWA and resolved on a 6% polyacrylamide gel. Competitor assays were performed by incubating the reaction with 100X excess of unlabeled (no biotin) oligonucleotide complexes with identical sequence. Arrow denotes specific binding of HWA nuclear protein to reference (left) and alternate (right) allele. (a) First replicate of the EMSA for rs4776984. (b) Second replicate of the EMSA for rs4776984.

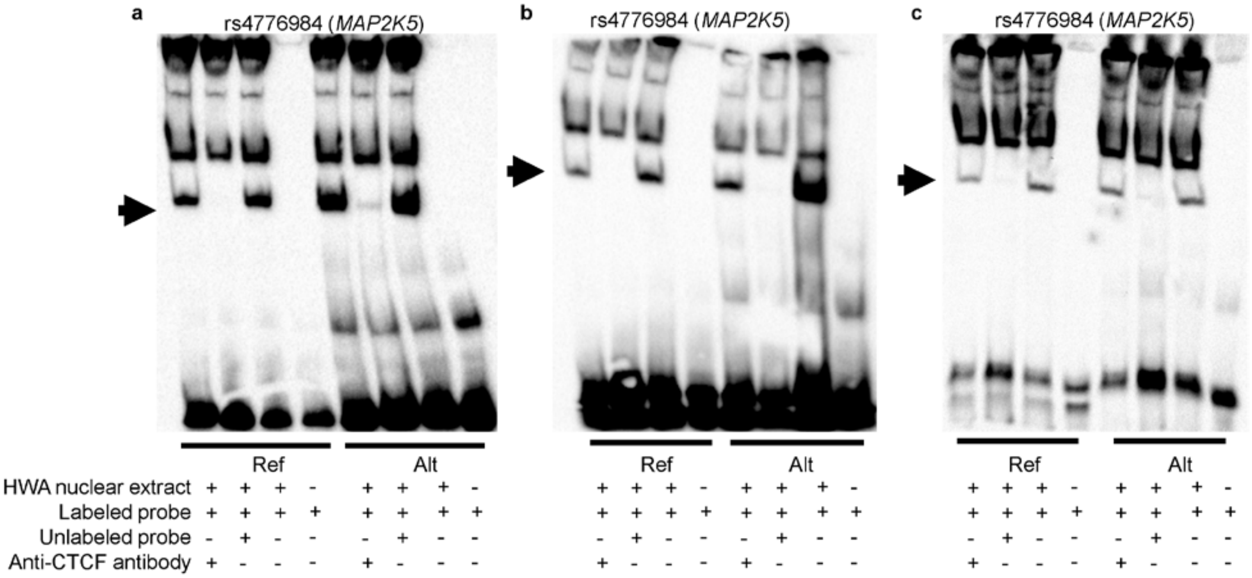

Supplementary Figure 5. Three independent replicates for the Electrophoretic mobility shift assay (EMSA) do not show a supershift when using antibody against CTCF and nuclear protein extracted from primary human white adipocytes (HWA) at the *MAP2K5* *cis*-eQTL SNP rs4776984.

Biotinylated (labeled probe) 31-bp oligonucleotide complexes with +/-15 bp flanking the reference or alternate allele for variant rs4776984 were incubated with nuclear protein extracted from primary HWA and resolved on a 6% polyacrylamide gel. Competitor assays were performed by incubating the reaction with 100X excess of unlabeled (no biotin) oligonucleotide complexes with identical sequence. Arrow denotes specific binding of HWA nuclear protein to reference (left) and alternate (right) allele. Supershift assays were performed with 1μg anti-CTCF antibodies (Santa Cruz sc-15914). (a) First replicate of the supershift EMSA for rs4776984. (b) Second replicate of the supershift EMSA for rs4776984. (c) Third replicate of the supershift EMSA for rs4776984.

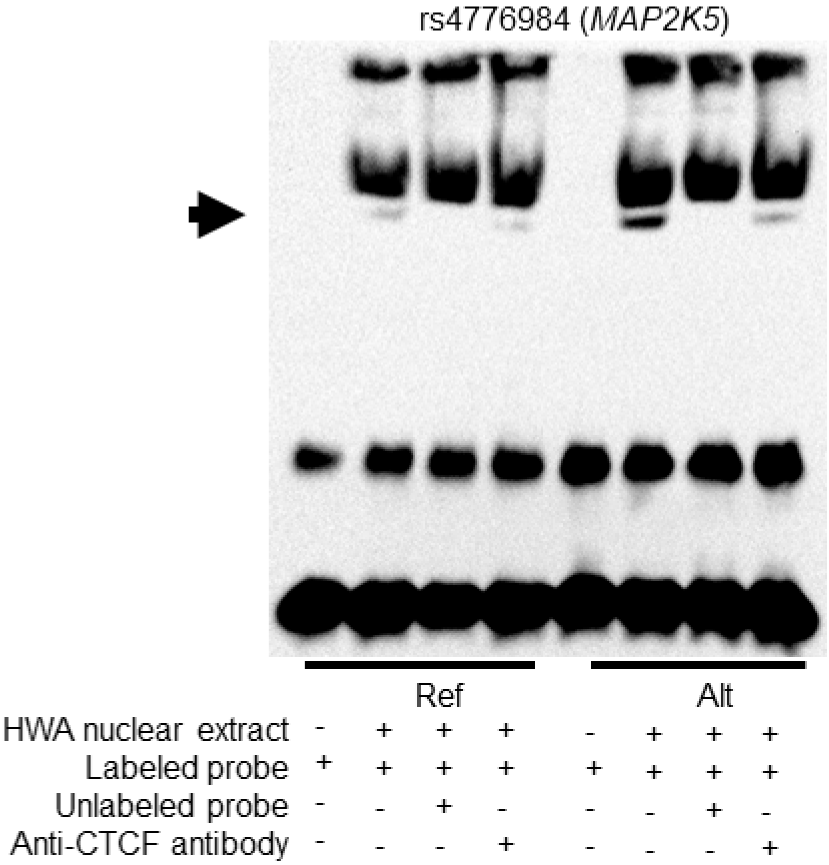

Supplementary Figure 6. The Electrophoretic mobility shift assay (EMSA) does not show a supershift when using a different antibody against CTCF and nuclear protein extracted from primary human white adipocytes (HWA) at the *MAP2K5 cis*-eQTL SNP rs4776984.

Biotinylated (labeled probe) 31-bp oligonucleotide complexes with +/-15 bp flanking the reference or alternate allele for variant rs4776984 were incubated with nuclear protein extracted from primary HWA and resolved on a 6% polyacrylamide gel. Competitor assays were performed by incubating the reaction with 100X excess of unlabeled (no biotin) oligonucleotide complexes with identical sequence. Arrow denotes specific binding of HWA nuclear protein to reference (left) and alternate (right) allele. Supershift assays were performed with 1μg anti-CTCF antibodies (EMD Millipore 07-729).

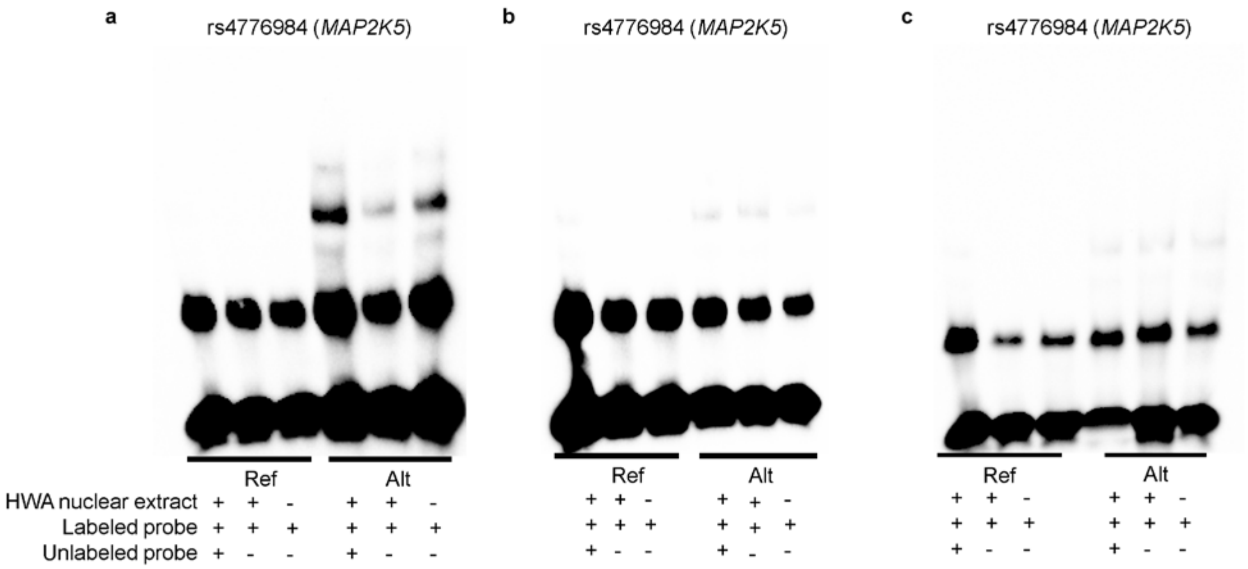

Supplementary Figure 7. Three independent replicates for the Electrophoretic mobility shift assay (EMSA) do not show specific binding using purified CTCF protein at the *MAP2K5 cis*-eQTL SNP rs4776984.

Biotinylated (labeled probe) 31-bp oligonucleotide complexes with +/-15 bp flanking the reference or alternate allele for variant rs4776984 were incubated with purified CTCF protein (Origene TP720882) and resolved on a 6% polyacrylamide gel in our EMSA experiment. Competitor assays were performed by incubating the reaction with 100X excess of unlabeled (no biotin) oligonucleotide complexes with identical sequence. The reference allele is on the left and alternate allele on the right. (a) First replicate of the EMSA for rs4776984. (b) Second replicate of the EMSA for rs4776984. (c) Third replicate of the EMSA for rs4776984.

Supplementary Table 1. Parameters used for identification of novel *cis*-eQTL and looping interactions

cis-eQTL discovery	METSIM (n=335)
Type of genetic data	Illumina Omni Express
# *cis*-eQTLSNPs with the same target gene and beta direction replicated in subcutaneous adipose GTEx data	386,068
# PEER factors corrected	22
# Genetic principal components corrected	3
Minor allele frequency (MAF)	> 5%
Type of expression data	RNA-seq
Normalization technique	Inverse normal transformation of FPKMs
FDR significance threshold for cis-eQTL SNPs	< 5%
# of *cis*-eQTL target genes with looping interactions	4,332
Promoter capture Hi-C	Primary human white adipocytes
# reads from sequencing	138,217,259
# uniquely aligned paired reads	101,187,918
# valid pairs of reads after capture Hi-C specific filtering by HiCUP	88,583,089
# significant looping interaction pairs identified from CHiCAGO	80,567
# METSIM genes in looping interaction pairs	10,083

Supplementary Table 2. Histone mark enrichment in looping *Hin*dIII fragments in primary HWA

Histone mark	Base pairs of feature enrichment in looping *Hin*dIII fragments	Base pairs of feature enrichment in random *Hin*dII fragments*	Standard deviation	*p*-value†
H3K4me1	42181	39278.45	56.94	$<2.2x10^{-16}$
H3K4me3	42347	39502.39	55.86	$<2.2x10^{-16}$
H3K27ac	42095	39597.19	49.24	$<2.2x10^{-16}$
H3K27me3	42813	40529.33	43.78	$<2.2x10^{-16}$
H3K9me3	41222	39408.19	54.07	$<2.2x10^{-16}$
DHS	35578	30547.74	89.82	$<2.2x10^{-16}$

*Random *Hin*dIII fragments were controlled for distance away from the target promoter when selected.

†*p*-value computed from Pearson's chi-squared test.

Supplementary Table 3. Adipocyte chromosomal interactions are enriched for 30 transcription factors (adjusted *p*<0.05) when compared to CD34+ chromosomal interactions

Motif logo	Motif name	*p*-value	Adjusted *p*-value	Number of target sequences with motif (of 189013)	Percent of target sequences with motif	Number of background sequences with motif (of 173261)	Percent of background sequences with motif
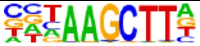	CTCF(Zf)/CD4+-CTCF-ChIP-Seq(Barski_et_al.)/Homer	$1.00x10^{-102}$	0	5746	3.04%	3915.7	2.26%
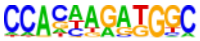	BORIS(Zf)/K562-CTCFL-ChIP-Seq(GSE32465)/Homer	$1.00x10^{-53}$	0	6336	3.35%	4760.1	2.75%
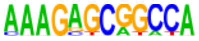	CEBP(bZIP)/ThioMac-CEBPb-ChIP-Seq(GSE21512)/Homer	$1.00x10^{-10}$	0	18925	10.01%	16547.8	9.56%
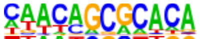	Sp5(Zf)/mES-Sp5.Flag-ChIP-Seq(GSE72989)/Homer	$1.00x10^{-7}$	0	16995	8.99%	14950.8	8.64%
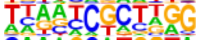	Elk4(ETS)/Hela-Elk4-ChIP-Seq(GSE31477)/Homer	$1.00x10^{-5}$	0.00010	10491	5.55%	9180.3	5.31%
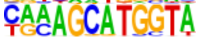	YY1(Zf)/Promoter/Homer	$1.00x10^{-5}$	0.00030	817	0.43%	638.7	0.37%
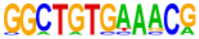	NRF1(NRF)/MCF7-NRF1-ChIP-Seq(Unpublished)/Homer	$1.00x10^{-5}$	0.00040	1163	0.61%	935.2	0.54%
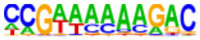	TEAD2(TEA)/Py2T-Tead2-ChIP-Seq(GSE55709)/Homer	$1.00x10^{-4}$	0.00050	12965	6.86%	11440.4	6.61%
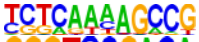	E2F3(E2F)/MEF-E2F3-ChIP-Seq(GSE71376)/Homer	$1.00x10^{-4}$	0.00070	7993	4.23%	6986.6	4.04%
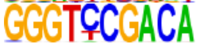	Erra(NR)/HepG2-Erra-ChIP-Seq(GSE31477)/Homer	$1.00x10^{-4}$	0.0023	40662	21.50%	36580.6	21.14%
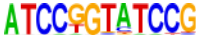	TEAD(TEA)/Fibroblast-PU.1-ChIP-Seq(Unpublished)/Homer	$1.00x10^{-4}$	0.0026	17798	9.41%	15848.7	9.16%
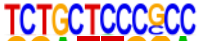	Elk1(ETS)/Hela-Elk1-ChIP-Seq(GSE31477)/Homer	$1.00x10^{-4}$	0.0026	10671	5.64%	9422.4	5.45%
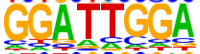	TEAD4(TEA)/Tropoblast-Tead4-ChIP-Seq(GSE37350)/Homer	$1.00x10^{-4}$	0.0026	20464	10.82%	18265.1	10.56%
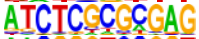	GFY(?)/Promoter/Homer	$1.00x10^{-3}$	0.0031	1159	0.61%	950.2	0.55%
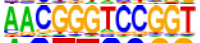	E2F4(E2F)/K562-E2F4-ChIP-Seq(GSE31477)/Homer	$1.00x10^{-3}$	0.0069	5131	2.71%	4475.5	2.59%
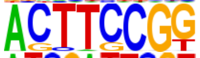	Sp1(Zf)/Promoter/Homer	$1.00x10^{-3}$	0.0084	3622	1.92%	3133.5	1.81%
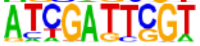	NFY(CCAAT)/Promoter/Homer	$1.00x10^{-3}$	0.0084	14237	7.53%	12676.1	7.33%
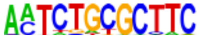	Ronin(THAP)/ES-Thap11-ChIP-Seq(GSE51522)/Homer	$1.00x10^{-3}$	0.012	443	0.23%	346.4	0.20%
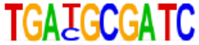	Olig2(bHLH)/Neuron-Olig2-ChIP-Seq(GSE30882)/Homer	$1.00x10^{-3}$	0.014	45873	24.26%	41429.3	23.94%
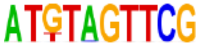	E2F6(E2F)/Hela-E2F6-ChIP-Seq(GSE31477)/Homer	$1.00x10^{-2}$	0.022	7047	3.73%	6221.3	3.60%
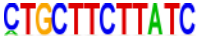	ZNF143|STAF(Zf)/CUTLL-ZNF143-ChIP-Seq(GSE29600)/Homer	$1.00x10^{-2}$	0.024	5580	2.95%	4906.1	2.84%
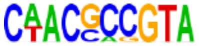	DUX4(Homeobox)/Myoblasts-DUX4.V5-ChIP-Seq(GSE75791)/Homer	$1.00x10^{-2}$	0.029	936	0.49%	777.7	0.45%
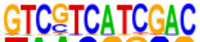	NRF(NRF)/Promoter/Homer	$1.00x10^{-2}$	0.036	1622	0.86%	1382.4	0.80%
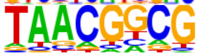	PPARE(NR),DR1/3T3L1-Pparg-ChIP-Seq(GSE13511)/Homer	$1.00x10^{-2}$	0.039	17385	9.19%	15586.6	9.01%
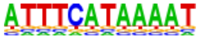	Pax7(Paired,Homeobox),longest/Myoblast-Pax7-ChIP-Seq(GSE25064)/Homer	$1.00x10^{-2}$	0.040	513	0.27%	415	0.24%
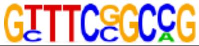	NFAT(RHD)/Jurkat-NFATC1-ChIP-Seq(Jolma_et_al.)/Homer	$1.00x10^{-2}$	0.045	20039	10.60%	18002.5	10.40%

Supplementary Table 4. LD score enrichments, heritability estimates, and *p*-values using the published LD Score software[9]

Category	Prop. of SNPs	Prop. of h^2	Enrichment	SE	*p*-value
Coding_UCSC	0.02	0.15	9.26	0.9	$6.66x10^{-23}$
Coding_UCSC.extend.500	0.07	0.28	4.21	0.3	$1.27x10^{-27}$
Conserved_LindbladToh	0.03	0.15	5.32	0.8	$1.26x10^{-8}$
Conserved_LindbladToh.extend.500	0.34	0.56	1.64	0.1	$4.45x10^{-7}$
CTCF_Hoffman	0.02	0.06	2.52	0.5	$4.92x10^{-3}$
CTCF_Hoffman.extend.500	0.07	0.11	1.58	0.3	$2.59x10^{-2}$
DGF_ENCODE	0.14	0.34	2.46	0.2	$6.87x10^{-10}$
DGF_ENCODE.extend.500	0.54	0.77	1.42	0.1	$2.60x10^{-5}$
DHS_peaks_Trynka	0.11	0.27	2.37	0.3	$3.92x10^{-6}$
DHS_Trynka	0.17	0.35	2.05	0.2	$8.32x10^{-6}$
DHS_Trynka.extend.500	0.50	0.72	1.44	0.1	$3.56x10^{-5}$
Enhancer_Andersson	0.00	0.01	2.60	2.0	$4.30x10^{-1}$
Enhancer_Andersson.extend.500	0.02	0.03	1.70	0.7	$2.87x10^{-1}$
Enhancer_Hoffman	0.06	0.15	2.27	0.3	$6.34x10^{-5}$
Enhancer_Hoffman.extend.500	0.16	0.30	1.93	0.2	$2.51x10^{-7}$
FetalDHS_Trynka	0.09	0.28	3.25	0.3	$3.78x10^{-12}$
FetalDHS_Trynka.extend.500	0.29	0.48	1.69	0.2	$2.28x10^{-8}$
H3K27ac_Hnisz	0.39	0.65	1.64	0.1	$2.77x10^{-8}$
H3K27ac_Hnisz.extend.500	0.43	0.68	1.58	0.1	$9.46x10^{-8}$
H3K27ac_PGC2	0.27	0.53	1.95	0.1	$2.57x10^{-11}$
H3K27ac_PGC2.extend.500	0.34	0.61	1.80	0.1	$8.44x10^{-1}$
H3K4me1_peaks_Trynka	0.18	0.35	1.98	0.2	$2.26x10^{-6}$
H3K4me1_Trynka	0.43	0.71	1.64	0.1	$5.61x10^{-8}$
H3K4me1_Trynka.extend.500	0.61	0.86	1.41	0.1	$6.55x10^{-6}$
H3K4me3_peaks_Trynka	0.04	0.11	2.55	0.6	$1.35x10^{-3}$
H3K4me3_Trynka	0.14	0.35	2.59	0.2	$5.48x10^{-13}$
H3K4me3_Trynka.extend.500	0.26	0.49	1.88	0.1	$4.52x10^{-10}$
H3K9ac_peaks_Trynka	0.04	0.12	3.03	0.5	$1.03x10^{-5}$
H3K9ac_Trynka	0.13	0.36	2.79	0.2	$1.07x10^{-14}$
H3K9ac_Trynka.extend.500	0.23	0.50	2.15	0.2	$1.18x10^{-14}$
Intron_UCSC	0.39	0.40	1.00	0.1	$9.63x10^{-1}$
Intron_UCSC.extend.500	0.40	0.51	1.27	0.1	$3.63x10^{-3}$
PromoterFlanking_Hoffman	0.01	0.04	4.43	1.1	$1.82x10^{-3}$
PromoterFlanking_Hoffman.extend.500	0.03	0.13	3.70	0.4	$3.39x10^{-12}$
Promoter_UCSC	0.03	0.14	4.45	0.5	$6.18x10^{-12}$
Promoter_UCSC.extend.500	0.04	0.18	4.51	0.4	$1.01x10^{-20}$
Repressed_Hoffman	0.45	0.30	0.65	0.1	$5.99x10^{-20}$
Repressed_Hoffman.extend.500	0.71	0.47	0.65	0.1	$8.43x10^{-6}$
SuperEnhancer_Hnisz	0.17	0.35	2.03	0.2	$1.79x10^{-6}$
SuperEnhancer_Hnisz.extend.500	0.17	0.34	1.97	0.2	$1.57x10^{-6}$
TFBS_ENCODE	0.13	0.34	2.50	0.2	$2.61x10^{-11}$
TFBS_ENCODE.extend.500	0.35	0.49	1.42	0.1	$6.98x10^{-4}$
Transcribed_Hoffman	0.35	0.38	1.07	0.1	$5.34x10^{-1}$
Transcribed_Hoffman.extend.500	0.77	0.74	0.97	0.1	$6.53x10^{-1}$
TSS_Hoffman	0.02	0.13	7.10	0.9	$5.15x10^{-12}$
TSS_Hoffman.extend.500	0.04	0.19	5.23	0.5	$6.73x10^{-15}$
UTR_3_UCSC	0.01	0.08	6.88	0.9	$4.11x10^{-10}$
UTR_3_UCSC.extend.500	0.03	0.13	4.48	0.6	$4.99x10^{-10}$
UTR_5_UCSC	0.01	0.05	9.46	1.8	$1.36x10^{-6}$
UTR_5_UCSC.extend.500	0.03	0.15	5.10	0.5	$1.19x10^{-14}$
WeakEnhancer_Hoffman	0.02	0.04	1.92	0.6	$1.02x10^{-1}$
WeakEnhancer_Hoffman.extend.500	0.09	0.17	1.92	0.2	$1.58x10^{-4}$

Supplementary Table 5. LD score enrichments, heritability estimates, and *p*-values after modification of the LD score software

Category	Prop. of SNPs	Prop. of h^2	Enrichment	SE	*p*-value
Coding_UCSC	0.03	0.20	6.64	0.6	$3.44x10^{-22}$
Coding_UCSC.extend.500	0.12	0.39	3.19	0.2	$5.65x10^{-27}$
Conserved_LindbladToh	0.03	0.16	4.82	0.5	$3.25x10^{-13}$
Conserved_LindbladToh.extend.500	0.40	0.63	1.59	0.1	$3.09x10^{-10}$
CTCF_Hoffman	0.03	0.07	2.18	0.4	$1.54x10^{-3}$
CTCF_Hoffman.extend.500	0.10	0.14	1.47	0.2	$7.92x10^{-3}$
DGF_ENCODE	0.18	0.40	2.19	0.2	$3.73x10^{-12}$
DGF_ENCODE.extend.500	0.64	0.83	1.29	0.1	$1.23x10^{-4}$
DHS_peaks_Trynka	0.14	0.31	2.32	0.2	$2.94x10^{-9}$
DHS_Trynka	0.20	0.40	1.99	0.2	$7.84x10^{-9}$
DHS_Trynka.extend.500	0.56	0.76	1.34	0.1	$2.98x10^{-5}$
Enhancer_Andersson	0.01	0.01	2.21	1.4	$3.80x10^{-1}$
Enhancer_Andersson.extend.500	0.03	0.04	1.48	0.5	$2.89x10^{-1}$
Enhancer_Hoffman	0.10	0.19	1.95	0.2	$2.96x10^{-5}$
Enhancer_Hoffman.extend.500	0.23	0.38	1.66	0.1	$5.12x10^{-7}$
FetalDHS_Trynka	0.11	0.31	2.92	0.2	$4.30x10^{-15}$
FetalDHS_Trynka.extend.500	0.34	0.53	1.58	0.1	$1.68x10^{-7}$
H3K27ac_Hnisz	0.54	0.75	1.39	0.1	$7.15x10^{-6}$
H3K27ac_Hnisz.extend.500	0.57	0.77	1.35	0.1	$2.25x10^{-5}$
H3K27ac_PGC2	0.37	0.62	1.67	0.1	$7.02x10^{-10}$
H3K27ac_PGC2.extend.500	0.46	0.71	1.54	0.1	$6.89x10^{-9}$
H3K4me1_peaks_Trynka	0.24	0.41	1.74	0.2	$4.82x10^{-7}$
H3K4me1_Trynka	0.55	0.80	1.44	0.1	$9.82x10^{-7}$
H3K4me1_Trynka.extend.500	0.74	0.92	1.25	0.1	$3.15x10^{-4}$
H3K4me3_peaks_Trynka	0.06	0.15	2.36	0.3	$6.40x10^{-5}$
H3K4me3_Trynka	0.20	0.46	2.29	0.2	$4.79x10^{-15}$
H3K4me3_Trynka.extend.500	0.35	0.60	1.71	0.1	$2.44x10^{-11}$
H3K9ac_peaks_Trynka	0.07	0.17	2.50	0.3	$2.10x10^{-6}$
H3K9ac_Trynka	0.21	0.48	2.30	0.2	$5.70x10^{-15}$
H3K9ac_Trynka.extend.500	0.36	0.64	1.77	0.1	$4.00x10^{-13}$
Intron_UCSC	0.47	0.43	0.91	0.1	$2.60x10^{-1}$
Intron_UCSC.extend.500	0.49	0.60	1.21	0.1	$4.05x10^{-3}$
PromoterFlanking_Hoffman	0.01	0.04	3.30	0.8	$2.11x10^{-3}$
PromoterFlanking_Hoffman.extend.500	0.05	0.15	2.91	0.3	$3.11x10^{-12}$
Promoter_UCSC	0.06	0.20	3.41	0.4	$2.98x10^{-12}$
Promoter_UCSC.extend.500	0.07	0.25	3.43	0.3	$2.08x10^{-20}$
Repressed_Hoffman	0.34	0.18	0.55	0.1	$2.20x10^{-7}$
Repressed_Hoffman.extend.500	0.56	0.32	0.57	0.1	$1.10x10^{-15}$
SuperEnhancer_Hnisz	0.27	0.43	1.63	0.2	$1.92x10^{-5}$
SuperEnhancer_Hnisz.extend.500	0.27	0.43	1.59	0.1	$2.07x10^{-5}$
TFBS_ENCODE	0.18	0.41	2.26	0.2	$2.21x10^{-15}$
TFBS_ENCODE.extend.500	0.43	0.60	1.38	0.1	$7.13x10^{-5}$
Transcribed_Hoffman	0.43	0.43	1.02	0.1	$7.98x10^{-1}$
Transcribed_Hoffman.extend.500	0.76	0.72	0.94	0.1	$3.15x10^{-1}$
TSS_Hoffman	0.03	0.19	5.34	0.6	$9.97x10^{-13}$
TSS_Hoffman.extend.500	0.06	0.26	3.97	0.4	$1.84x10^{-15}$
UTR_3_UCSC	0.02	0.10	5.06	0.6	$2.30x10^{-10}$
UTR_3_UCSC.extend.500	0.05	0.16	3.41	0.4	$2.80x10^{-10}$
UTR_5_UCSC	0.01	0.07	7.15	1.2	$2.72x10^{-7}$
UTR_5_UCSC.extend.500	0.05	0.20	3.99	0.4	$4.37x10^{-16}$
WeakEnhancer_Hoffman	0.03	0.05	1.67	0.4	$7.95x10^{-2}$
WeakEnhancer_Hoffman.extend.500	0.13	0.22	1.62	0.2	$3.10x10^{-4}$

Supplementary Table 6. Fifty-four eGenes in METSIM, including the 42 genes replicated for correlation with BMI and effect direction in TwinsUK

		Pearson		Linear regression					
		METSIM†		METSIM‡			TwinsUK§		
Gene	Chr#	Effect size (r)	p-value	Effect size (β)	SE	p-value	Effect size (β)	SE	p-value
ADH1B	4	-0.45	$7.40x10^{-18}$	-0.21	0.02	$1.68x10^{-20}$	-0.58	0.03	$4.47x10^{-71}$
*ORMDL3**	17	-0.45	$8.57x10^{-18}$	-0.16	0.02	$2.06x10^{-20}$	-0.58	0.03	$2.65x10^{-70}$
AKR1C3	10	0.33	$4.78x10^{-10}$	0.13	0.02	$2.95x10^{-11}$	0.49	0.03	$5.19x10^{-54}$
CMTM3	16	0.41	$4.32x10^{-15}$	0.087	0.01	$3.84x10^{-17}$	0.50	0.03	$6.64x10^{-52}$
LPIN1	2	-0.38	$1.49x10^{-13}$	-0.14	0.02	$2.27x10^{-15}$	-0.47	0.03	$2.38x10^{-44}$
RNF157	17	-0.29	$5.19x10^{-8}$	-0.096	0.02	$5.87x10^{-9}$	-0.47	0.03	$8.86x10^{-42}$
MYOF	10	0.32	$1.07x10^{-9}$	0.086	0.01	$7.37x10^{-11}$	0.46	0.03	$2.59x10^{-40}$
NAA40	11	0.28	$1.81x10^{-7}$	0.052	0.009	$2.67x10^{-8}$	0.46	0.03	$4.00x10^{-40}$
TMEM165	4	0.33	$2.45x10^{-9}$	0.045	0.007	$1.84x10^{-10}$	0.45	0.03	$3.52x10^{-37}$
RFFL	11	0.27	$1.02x10^{-6}$	0.035	0.006	$1.84x10^{-8}$	0.43	0.03	$5.67x10^{-37}$
TMCO6	5	-0.28	$9.23x10^{-8}$	-0.060	0.01	$1.18x10^{-8}$	-0.44	0.03	$5.04x10^{-35}$
SCRN2	17	-0.38	$2.23x10^{-13}$	-0.10	0.01	$3.79x10^{-15}$	-0.38	0.03	$5.32x10^{-35}$
CSGALNACT1	8	0.24	$1.00x10^{-5}$	0.047	0.01	$2.04x10^{-6}$	0.42	0.03	$1.41x10^{-31}$
TAPBP	6	0.25	$6.71x10^{-6}$	0.047	0.02	$1.60x10^{-6}$	0.32	0.03	$1.52x10^{-29}$
CLN8	8	0.32	$4.50x10^{-9}$	0.044	0.007	$3.67x10^{-10}$	0.36	0.03	$4.41x10^{-29}$
DRAM1	12	0.30	$1.87x10^{-8}$	0.050	0.008	$1.80x10^{-9}$	0.40	0.03	$5.94x10^{-29}$
WNT2B	1	0.25	$2.44x10^{-6}$	0.026	0.005	$4.90x10^{-7}$	0.38	0.03	$1.41x10^{-27}$
S100A1	1	-0.27	$2.52x10^{-7}$	-0.20	0.04	$3.59x10^{-8}$	-0.38	0.03	$3.69x10^{-26}$
RPS6KL1	14	0.26	$2.54x10^{-6}$	0.060	0.01	$5.25x10^{-7}$	0.34	0.03	$3.27x10^{-25}$
SLC16A7	12	-0.26	$3.47x10^{-6}$	-0.068	0.01	$7.60x10^{-7}$	-0.30	0.03	$2.08x10^{-23}$
ZNF592	15	-0.27	$8.26x10^{-7}$	-0.037	0.007	$1.40x10^{-7}$	-0.33	0.03	$2.10x10^{-23}$
MFSD1	3	0.31	$8.31x10^{-9}$	0.069	0.01	$6.70x10^{-10}$	0.35	0.04	$2.82x10^{-22}$
HYI	1	-0.31	$6.45x10^{-9}$	-0.11	0.02	$5.52x10^{-10}$	-0.29	0.03	$5.95x10^{-22}$
ANXA4	2	0.24	$1.04x10^{-5}$	0.045	0.009	$2.52x10^{-6}$	0.35	0.04	$1.20x10^{-21}$
RAB30	11	0.24	$8.19x10^{-6}$	0.040	0.008	$1.98x10^{-6}$	0.31	0.03	$1.16x10^{-20}$
PLD1	3	-0.28	$2.26x10^{-7}$	-0.050	0.009	$3.24x10^{-8}$	-0.32	0.03	$7.95x10^{-20}$
MYO5A	15	0.30	$3.20x10^{-8}$	0.049	0.008	$3.41x10^{-9}$	0.32	0.04	$4.61x10^{-19}$
*ACADS**	12	-0.37	$2.91x10^{-12}$	-0.085	0.01	$7.12x10^{-14}$	-0.24	0.03	$6.65x10^{-19}$
SCAI	9	-0.28	$1.81x10^{-7}$	-0.034	0.006	$2.50x10^{-8}$	-0.27	0.03	$1.42x10^{-18}$
HLA-DRB1	6	0.25	$3.53x10^{-6}$	0.14	0.03	$7.83x10^{-7}$	0.31	0.03	$2.09x10^{-18}$
*LACTB**	15	0.30	$1.67x10^{-8}$	0.069	0.01	$1.40x10^{-9}$	0.32	0.04	$4.94x10^{-18}$
GPHN	14	-0.43	$7.51x10^{-17}$	-0.11	0.01	$3.20x10^{-19}$	-0.29	0.03	$4.28x10^{-17}$
MPHOSPH8	13	-0.24	$8.25x10^{-6}$	-0.033	0.007	$2.02x10^{-6}$	-0.23	0.04	$3.97x10^{-11}$
*MAP2K5**	15	-0.25	$7.83x10^{-6}$	-0.039	0.008	$1.90x10^{-6}$	-0.21	0.03	$3.81x10^{-10}$
RRNAD1	1	-0.24	$1.05x10^{-5}$	-0.032	0.007	$2.30x10^{-6}$	-0.19	0.03	$3.14x10^{-9}$
CCDC50	3	-0.33	$1.16x10^{-9}$	-0.059	0.009	$7.24x10^{-11}$	-0.18	0.03	$9.93x10^{-9}$
RAD54L2	3	-0.25	$2.32x10^{-6}$	-0.030	0.006	$4.70x10^{-7}$	-0.20	0.04	$2.78x10^{-8}$
SCMH1	1	-0.32	$1.11x10^{-9}$	-0.047	0.007	$7.55x10^{-11}$	-0.19	0.03	$3.85x10^{-8}$
ATP7B	13	-0.26	$6.30x10^{-7}$	-0.040	0.007	$1.11x10^{-7}$	-0.20	0.04	$7.22x10^{-8}$
CYP7B1	8	0.24	$6.90x10^{-6}$	0.047	0.01	$1.64x10^{-6}$	0.19	0.03	$1.07x10^{-7}$
RERE	1	-0.24	$1.02x10^{-5}$	-0.031	0.006	$2.61x10^{-6}$	-0.17	0.04	5.39x10-6
RPAP1	15	-0.35	$1.86x10^{-10}$	-0.042	0.006	$8.82x10^{-12}$	-0.14	0.03	$9.58x10^{-6}$
ARHGEF7	13	-0.35	$4.12x10^{-11}$	-0.050	0.007	$1.60x10^{-12}$	0.022	0.04	NS‖
NCKIPSD	3	-0.34	$3.28x10^{-10}$	-0.067	0.01	$1.51x10^{-11}$	-0.042	0.03	NS‖
NDUFS2	1	-0.24	$9.38x10^{-6}$	-0.029	0.006	$2.17x10^{-6}$	-0.048	0.03	NS‖
REEP1	2	-0.24	$6.38x10^{-6}$	-0.033	0.007	$1.45x10^{-6}$	0.022	0.04	NS‖
RGCC	13	-0.25	$2.81x10^{-6}$	-0.076	0.01	$4.91x10^{-7}$	0.087	0.03	NS‖
SETD6	16	-0.27	$3.99x10^{-7}$	-0.041	0.007	$6.30x10^{-8}$	-0.047	0.04	NS‖
SLC35A3	12	-0.26	$1.13x10^{-6}$	-0.024	0.004	$2.15x10^{-7}$	0.043	0.03	NS‖
SPAG7	17	-0.26	$1.21x10^{-6}$	-0.035	0.007	$2.27x10^{-7}$	-0.076	0.03	NS‖
NUDCD3	7	-0.34	$7.00x10^{-10}$	-0.032	0.005	$4.17x10^{-11}$	NA¶	NA¶	NA¶
RP11-387H17.4	17	-0.40	$4.40x10^{-14}$	-0.26	0.03	$4.74x10^{-16}$	NA¶	NA¶	NA¶
RSBN1L-AS1	7	-0.36	$1.65x10^{-11}$	-0.056	0.007	$5.73x10^{-13}$	NA¶	NA¶	NA¶
TUBB2B	6	0.34	$1.01x10^{-10}$	0.14	0.02	$4.51x10^{-12}$	NA¶	NA¶	NA¶

*GWAS gene.
†Effect size (r, Pearson rho) and *p*-value calculated from Pearson correlation between gene expression and BMI (see Methods).
‡ Effect size, standard error (SE), and *p*-value calculated using a linear regression model with BMI and age, age^2 and the 14 technical factors as co-variates when compared to a null model without BMI. These models were compared using an F-test (see Methods).
§Effect size, standard error (SE), and *p*-value calculated from linear mixed effects model. A full model including BMI was compared to a null model in which the same model was fitted, but with the phenotype (BMI) omitted. These models were compared using an F-test (see Methods).
‖Adjusted p-value > $9.26x10^{-4}$.
¶Value not applicable due to inability to test for replication in TwinsUK cohort.
#Chr indicates chromosome.

Supplementary Table 7. The 42 replicated BMI-correlated eGenes show significant enrichment for metabolic and inflammatory pathways using KEGG pathway analysis as implemented in WebGestalt[13]

KEGG Pathway Name	Ratio of Enrichment	Number of Genes	Genes in Pathway	*p*-value	Adjusted *p*-value*
Fatty acid metabolism	18.76	2	*ACADS* *ADH1B*	0.0051	0.010
Metabolism of xenobiotics by cytochrome P450	21.78	2	*AKR1C3* *ADH1B*	0.0038	0.010
Steroid hormone biosynthesis	30.69	2	*AKR1C3* *CYP7B1*	0.0019	0.010
Antigen processing and presentation	11.85	2	*HLA-DRB1* *TAPBP*	0.012	0.019

**p*-value adjusted using Benjamini-Hochberg correction for multiple testing.

Supplementary Table 8. DeepSEA analysis of the variants in the MAP2K5 locus supports the functionality of the looping *cis*-eQTL SNP rs4776984.

SNP ID	Chr	Position	Ref	Alt	DeepSEA score
rs4776984	chr15	68118194	A	C	$2.36x10^{-3}$
rs4776982	chr15	68114974	A	G	$3.90x10^{-2}$
rs4492996	chr15	68113240	A	G	$7.16x10^{-2}$
rs4776990	chr15	68137364	C	T	$1.09x10^{-1}$
rs28742003	chr15	68127769	C	T	$1.30x10^{-1}$
rs28427879	chr15	68124256	G	T	$1.98x10^{-1}$

Supplementary Table 9. Significant CHiCAGO interaction and replication scores from a separate HWA Capture Hi-C experiment verify the looping *cis*-eQTLs for the four identified obesity-related loci.

Other End	Baited Fragment	Target Gene	Looping *cis*-eQTL	CHiCAGO score	Replication score
chr15,67834655,67840760	chr15,68111739,68138337	MAP2K5	rs4476984	5.05	6.15
chr17,38082534,38106859	chr17,38074576,38081958	ORMDL3	rs8076131	6.35	6.73
chr15,63413071,63415370	chr15,63561331,63570763	LACTB	rs3784671	6.65	13.92
chr12,121158545,121162946	chr12,121343847,121345146	ACADS	rs10774569	5.29	6.62

Supplementary Table 10. DNA oligonucleotides used for electrophoretic mobility shift assay.

DNA oligonucleotides	Sequence (5’ -> 3’) for positive and negative strand
Reference allele – A (positive) biotinylated probe*	GCGCGCCCAACTCGGAGCGCCCTGCTGGGCG
Reference allele – A (negative) biotinylated probe	CGCCCAGCAGGGCGCTCCGAGTTGGGCGCGC
Alternate allele – C (positive) biotinylated probe*	GCGCGCCCAACTCGGCGCGCCCTGCTGGGCG
Alternate allele – C (negative) biotinylated probe	CGCCCAGCAGGGCGCGCCGAGTTGGGCGCGC

*Biotinylated probes were created by adding biotin to the 5' end of positive strand probes.
